# Response to the letter to the Editor regarding “Treatment of infantile idiopathic scoliosis using a novel thoracolumbosacral orthosis: a case report”

**DOI:** 10.1186/s13256-022-03641-y

**Published:** 2022-12-05

**Authors:** Jeb McAviney, Benjamin T. Brown

**Affiliations:** 1 ScoliCare, Kogarah, Australia; 2 Department of Chiropractic, Faculty of Medicine, Health and Human Sciences, Macquarie University, Macquarie Park, Australia

We thank Fludder and Keil for highlighting the typographical error in the manuscript. A diagnosis of scoliosis is confirmed when the Cobb angle is ≥ 10° [1].

With regard to plagiocephaly, the early literature on infantile idiopathic scoliosis highlighted an association between scoliosis and plagiocephaly. In this field, the side of plagiocephaly has historically been classified according to the recessed area of the skull when viewing/measuring the patient’s skull from an anterior to posterior direction. The side of plagiocephaly according to this classification system is almost exclusively seen on the side of curve convexity [2, 3]. We have used this naming convention.

Fludder and Keil have suggested alternative options for the assessment and management of the patient in this case. These suggestions fail to account for the real-world context in which the case occurred. For example, an orthopedic surgeon, with knowledge of the previous examination findings, did perform an examination of the patient and did not recommend that magnetic resonance imaging be taken.

We believe this error in analysis is also reflected in some of the literature that Fludder and Keil have chosen to support their assertions. The paper on the utility of superficial abdominal reflexes in the diagnosis of scoliosis by Fujimori *et al.* [4] is one such example. This paper is a retrospective review of 93 patients (which included only 1 infant) who were scheduled for corrective surgery in a tertiary care setting. We would caution against attempting to apply diagnostic test accuracy statistics derived from a highly specialized patient population in a tertiary care setting to a primary care setting such as the one in our case. Studies that include more representative samples would strengthen Fludder and Keil’s argument.

There are inherent strengths and weakness associated with a case study design. We have provided the reader with all relevant information to judge the case presentation and have been transparent with the limitations associated with this case study.

Sincerely,

Jeb McAviney and Benjamin T. Brown

## Data Availability

Not applicable.
